# Using Consumer Perceptions of a Voice-Activated Speaker Device as an Educational Tool

**DOI:** 10.2196/17336

**Published:** 2020-04-24

**Authors:** Siubak Chung, Benjamin KP Woo

**Affiliations:** 1 University of California, Los Angeles Sylmar, CA United States

**Keywords:** consumer perceptions, voice-activated speaker device

## Abstract

Voice-activated smart speakers, with their ease of setup, low cost, and versatility, could be an affordable and accessible way to improve health and mental health outcomes. In 2018, there were a total of 320 comments generated from verified purchases of a voice-activated smart speaker. These comments revealed there could be potential benefits of reducing loneliness and social isolation for adult users, especially for the older population. However, further research is warranted to determine whether using such devices would be harmful to children’s physical or mental development.

## Introduction

Voice-activated speaker devices have recently gained popularity with the release of commercial products such as Amazon Echo and Google Home. In the United States, Amazon Echo and Google Home were released in 2014 and 2016, respectively. In China, Xiaomi launched a Chinese-language, voice-activated smart speaker, XiaoAI, in July 2017. XiaoAI has functionalities such as checking the weather, controlling smart home devices, playing music, and translating foreign languages [1]. In the second quarter of 2018, 2 million units of XiaoAI Speaker Mini were sold [2]. As voice-activated smart speakers are easy to set up, low-cost, and versatile, they could be an affordable and accessible way of improving health and mental health outcomes. At the same time, it should be noted that recent studies have documented the deficits of various artificial intelligence-powered voice assistants in responding to questions about interpersonal violence, mental health, and physical health [3,4].

Customer reviews from verified buyers on electronic commerce websites such as Amazon.com (United States) and Taobao.com (China) could help potential buyers learn more about a product. These customer reviews could also assist medical students and health professionals in understanding how technology could impact the mental health of device users. Therefore, we evaluated Taobao customer reviews, written in simplified Chinese, of a Xiaomi XiaoAI voice-activated smart speaker to gain a better understanding of the users of this type of technology.

## Methods

Leveraging user-generated textual data in the form of Taobao.com verified purchase reviews of a Xiaomi XiaoAI Speaker Mini in 2018, a retrospective review was performed. The recorded parameters included the number of verified purchase reviewer comments and the number of likes and dislikes. Among a total of 320 comments from verified buyers in 2018, there were 299 likes and 19 dislikes. The content of the positive and critical comments was also analyzed qualitatively. Negative comments regarding the weaknesses and defects of the device were excluded from this analysis.

## Results

Four comments indicated how the voice-activated speaker device could potentially impact mental health for adult users. For example, one reviewer wrote:

Having her [Xiaomi XiaoAI], I am no longer lonely. But sometimes she ignores me, and I don’t know why...But having her, I am no longer lonely.

Five comments suggested that the smart speaker may decrease loneliness and social isolation for older users. For example, an elderly user wrote:

My son came back home to install [Xiaomi XiaoAI] for me...Now my grandson wants to come to my house all the time, and he will sing with me and XiaoAI.

Moreover, there were an overwhelming 34 comments that highlighted the benefits of the voice-activated speaker device for children. For example, one parent wrote:

Xiaomi XiaoAI can speak to my child all day!

Another parent remarked:

My child loves it, and they need to create one for outdoor use.

Nevertheless, there was one parent who warned against its use:

Please do not buy for your child. Why do you want your child to talk to a dead object every minute, every second?

## Discussion

### Principal Findings

This preliminary qualitative research into consumer comments provides insight into consumers’ perception of voice-activated smart speakers for Chinese-language users. Our analysis revealed that there could be potential benefits including the reduction of loneliness and social isolation for Chinese-speaking adult users, especially for the older population. Although Chinese parents seem to acknowledge the benefits of having voice-activated speaker devices at home, further research is warranted to determine whether using such devices is harmful to children’s physical or mental development. Nevertheless, no matter the age group, users of smart speakers should practice moderation. Previous research studies have highlighted the advantages and disadvantages of social media and technology for Chinese-speaking participants [5-8]. Future studies could explicitly focus on the advantages and disadvantages of using voice-activated smart speakers among Chinese-speaking individuals of different age groups.

### Limitations and Future Directions

A few limitations should be considered when interpreting the results of this viewpoint. As noted, this viewpoint was preliminary and exploratory in nature. It only indicated the feasibility of using customer reviews to further understand a voice-activated smart speaker. In addition, some of the consumer comments were not from the direct users. As such, the comments from parents could be biased. Another limitation of this viewpoint was the exclusion of negative comments for the analysis. This preliminary viewpoint excluded negative comments because they mainly focused on the weaknesses and mechanical defects of the device. Further research is needed to determine the potential downsides of voice-activated smart speakers on mental health and mental health care. The use of focus groups in future studies could provide insights into the negative health and mental effects of such devices. Finally, future research could focus on how the presence of voice-activated speaker devices may enhance student learning. Medical students and trainees need to have a deeper understanding of how technology will change the future of health care, and understanding consumer perceptions of such products could be a way to supplement medical education for future physicians and health care professionals.
